# 

**Published:** 1917-05-05

**Authors:** 


### ST. THOMAS'S HOSPITAL.

House Appointments.—The following gentlemen have been selected as house officers from Tuesday, May 8, 1917: *Casualty Officers and Resident Anæsthetists.*—W. G. Woolrich, B.A. Cantab., M.R.C.S., L.R.C.P.; A. H. Pearce, B.A. Cantab., M.R.C.S., L.R.C.P.; L. B. Hartley, B.A. Cantab., M.R.C.S., L.R.C.P.; P. G. S. Davis, M.R.C.S., L.R.C.P. *Resident House Physicians.*—W. T. Beswick, B.A. Cantab., M.R.C.S., L.R.C.P.; A. Mavrogordato, M.A. Cantab., M.R.C.S., L.R.C.P.; T. Anwyl-Davies, M.R.C.S., L.R.C.P.; L. G. Higgins, B.A. Cantab. *Resident House Surgeons.*—J. L. Gilks, F.R.C.S. Edin., M.R.C.S., L.R.C.P.; H. C. Jennings, M.R.C.S., L.R.C.P., H. W. Leatham, B.A. Cantab., M.R.C.S., L.R.C.P.; J. Rowland, M.R.C.S., L.R.C.P. *House Surgeon to Block* 8.—W. Marriott, M.R.C.S., L.R.C.P. *Obstetric House Physician.*—C. V. Pink, M.R.C.S., L.R.C.P.

### Matrons' and Sisters' Appointments.

Royal Berkshire Hospital, Reading.—Miss Jeannie Brown has been appointed assistant matron. She was trained at Preston Royal Infirmary, where she was later sister and temporary assistant matron, and at Monsall Fever Hospital, Manchester, where she has since been ward sister.

Royal Surrey County Hospital, Guildford.—Miss Rosetta M. Jackson has been appointed sister. She was trained at the London Temperance Hospital, and has been staff nurse at Horton War Hospital, Epsom.

City Hospital, West Heath, Birmingham.—Miss Julia Lund has been appointed day sister. She was trained at Keighley Union Infirmary, and since her training has held the post of sister at Grimsby Union Infirmary, Rotherham Union Infirmary, City Hospital, Dingle, Liverpool, and Romsley Hill Sanatorium, Halesowen, near Birmingham. She has also done private nursing.

**NOTE.—Particulars of Appointments for insertion in this column will be welcomed.**

Rates of Subscription: Twelve months, 6/6; Six months, 4/-; Three months, 2/-; post free to any address in the United Kingdom. Abroad, Twelve months, 8/8; Six months, 5/-; Three months, 2/6, post free. The Hospital "Index" to Volume LX., April to September 1916, is now ready, price 2½d. per copy, post free. Address The Manager, "The Hospital," The Hospital Building, 28/29 Southampton Street, Strand, London, W.C. 2.

28/29 Southampton Street, Strand, London, W.C. 2.

